# Targeting of replicating CD133 and OCT4/SOX2 expressing glioma stem cells selects a cell population that reinitiates tumors upon release of therapeutic pressure

**DOI:** 10.1038/s41598-019-46014-0

**Published:** 2019-07-02

**Authors:** Marta Guerra-Rebollo, Cristina Garrido, Lourdes Sánchez-Cid, Carolina Soler-Botija, Oscar Meca-Cortés, Nuria Rubio, Jerónimo Blanco

**Affiliations:** 1 Cell Therapy Group, Institute for Advanced Chemistry of Catalonia - Spanish National Research Council (IQAC-CSIC), Jordi Girona, 18–26, Barcelona, 08034 Spain; 2 Networking Research Center on Bioengineering, Biomaterials and Nanomedicine (CIBER-BBN), Zaragoza, 50018 Spain; 3 ICREC (Heart Failure and Cardiac Regeneration) Research Program, Health Sciences Research Institute Germans Trias i Pujol (IGTP), Badalona, Barcelona Spain; 4 CIBER-CV, Instituto de Salud Carlos III, Madrid, Spain

**Keywords:** Cancer imaging, Cancer stem cells

## Abstract

The existence of radio- and chemotherapy-surviving cancer stem cells is currently believed to explain the inefficacy of anti-glioblastoma (GBM) therapies. The aim of this study was to determine if a therapeutic strategy specifically targeting GBM stem cells (GSC) would completely eradicate a GBM tumor. In both the *in vitro* and the *in vivo* models, ganciclovir therapy targeting proliferating GSC promotes the survival of a quiescent, stem-like cell pool capable of reproducing the tumor upon release of the therapeutic pressure. Images of small niches of therapy-surviving tumor cells show organized networks of vascular-like structures formed by tumor cells expressing CD133 or OCT4/SOX2. These results prompted the investigation of tumor cells differentiated to endothelial and pericytic lineages as a potential reservoir of tumor-initiating capacity. Isolated tumor cells with pericyte and endothelial cell lineage characteristics, grown under tumorsphere forming conditions and were able to reproduce tumors after implantation in mice.

## Introduction

Glioblastoma (GBM) is the most frequent primary malignant brain tumor. Despite advances in treatment, GBM therapy is still focused on surgical resection followed by radiation and chemotherapy and its prognosis remains poor; the median survival for GBM patients is only 12 to 15 months^1^. Although GBM rarely metastasizes to distant organs, the tumor often invades surrounding regions of the brain which makes complete surgical resection impossible^2^.

At the histological level, GBMs show a cellular hierarchy dominated by stem-like cells^3,4^. Glioblastoma stem cells (GSCs), at the apex of this hierarchy, are functionally defined as having the capacity for self-renewal, multipotent differentiation and recapitulation of the original tumor phenotype, upon implantation in immunodeficient mice^5,6^. Clinically relevant, GSCs appear to be highly resistant to radio- and chemotherapy, a fact that may contribute to explain the inefficiency of standard tumor therapies^7,8^.

While not exclusive of GBM, GSCs can be characterized by the expression of a variety of markers within the class of transcription factors such as SOX2^9^, OCT4^9^, NANOG^10^, OLIG2^11^, NESTIN^12^, ID1^13^, and cell surface proteins such as CD133^14^, CD15^15^, CD44^14,16^, LICAM^17^ or A2B5^18^.

Emerging studies show that the cancer stem-like phenotype is regulated by different autocrine/paracrine and environmental signals and that differentiated cancer progenitor cells have the capacity to dedifferentiate and acquire a stem-like phenotype in response to these external stimuli^19,20^.

GSC can reside at least in two different niches within the same tumor: hypoxic and perivascular niches. The hypoxic niche plays a key regulatory role for the GSC phenotype, either by directly inducing the expression of self-renewal genes or by regulating cell differentiation^21^. GSCs are also found next to capillaries associated with endothelial cells^22,23^. GSCs promote the formation of tumor blood vessels, through the release of vascular endothelial growth factor (VEGF)^24^, generating the niche that supports its self-maintenance^25^.

Different studies support the notion that GSCs can give rise to vascular structures by differentiating into endothelial cells that form blood vessels^26,27^ and pericytes that support vessel function and tumor growth^28^ or via vasculogenic mimicry, where tumor cells form a fluid-conducting matrix through the acquisition of plasticity that mimics endothelial function^29,30^.

We had determined previously that adipose mesenchymal stem cells (AMSCs) modified to express the HSV thymidine kinase gene when implanted in GBM tumors differentiate to the endothelial lineage and associate with tumor vasculature structures and with CD133+ cancer stem cells. Treatment with the prodrug ganciclovir (GCV) effectively targeted replicating tumor cells reducing tumor cell burden by a factor of 10.000 and increasing animal survival^23,31^. However, while tumors could become chronic by periodic therapeutic cell administration, complete tumor cure was infrequent and tumors eventually relapsed. Thus, we speculated that the therapeutic effectiveness resulted from targetting the GSC vascular niche.

In the current work, we seek to determine if a therapeutic strategy specifically designed to target GSCs in a GBM model could completely eradicate a glioblastoma tumor. We show that even after extended treatment against replicating GSCs, a pool of resistant tumor cells is selected that can reproduce the tumors upon release from the therapeutic pressure. Moreover, we identify in the tumor vascular compartment tumor cells that express endothelial and pericytic markers and are endowed with the capacity to recapitulate the tumor.

## Results

### CD133 and OCT4/SOX2 promoters regulated RFP and Renilla luciferase reporters of GBM stemmess *in vitro*

Human GBM U87 tumor cells were genetically modified by transduction with a lentiviral vector construct for stable expression of a chimeric reporter comprising *Photinus pyralis* luciferase (PLuc) and enhanced green fluorescence protein (eGFP) activities, controlled by the cytomegalovirus (CMV) constitutive promoter.

**Figure 1:**
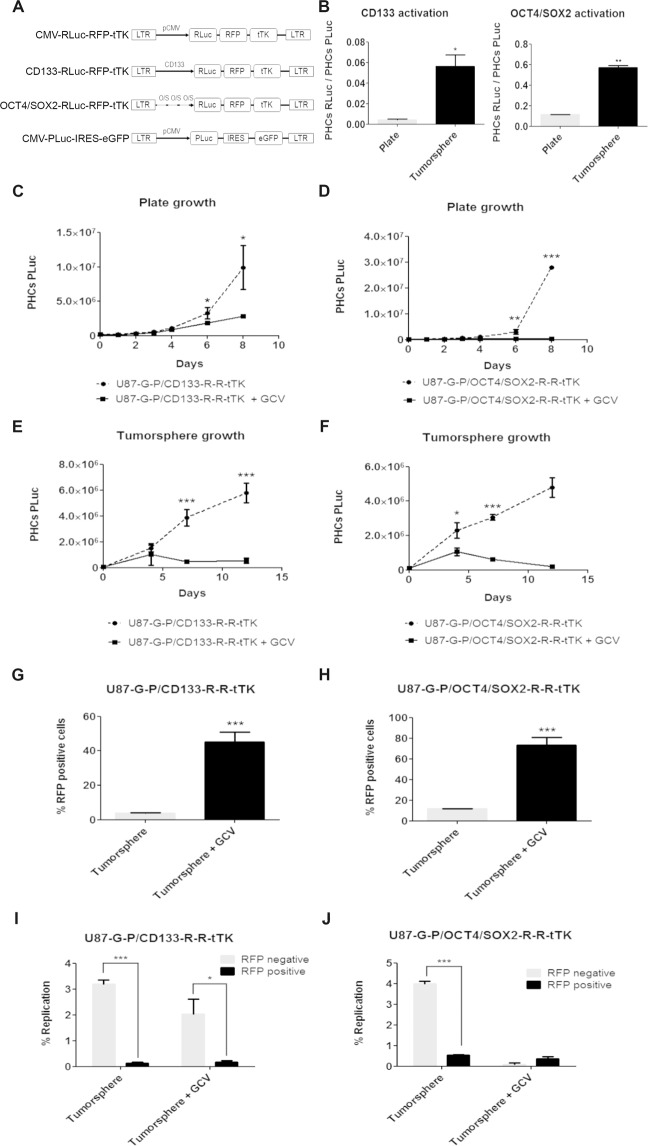
Gene constructs and functionality tests. (**A)** Diagrams showing the luciferase reporters constructs used for transduction of U87 cells: CMV-RLuc-RFP-tTK, CD133-RLuc‐RFP‐tTK, OCT4/SOX2-RLuc‐RFP‐tTK, CMV-PLuc-IRES-eGFP. **(B)** Histograms showing changes in the ratio of RLuc/PLuc activity in response to growth conditions; **(C**–**F**) Graphs show the effect of GCV (4 µg/ml) on cell growth. PHCs correspond to PLuc photon counts from BLI images; **(G**,**H)** Flow cytometry analysis of GCV treated cells showing changes in the fraction of red fluorescent cells; **(I**,**J)** Graphs showing the fraction of replicating cells in RFP negative and positive cells, before and after the GCV treatment. Data points show the average of sextuplicate measurements; bars, standard deviation of the mean; *P < 0.5, **P < 0.01, ***P < 0.0001.

In addition, FACS-selected eGFP positive cells were transduced with a second trifunctional chimeric reporter for expression of *Renilla reniformis* luciferase (RLuc), the red fluorescent protein (RFP) and a truncated version of the herpes simplex virus thymidine kinase sr39tk (tTK) under control of either the CD133 or the OCT4/SOX2 promoters (Fig. 1A). This strategy allowed independent monitoring by bioluminescence imaging (BLI) and confocal microscopy, of either the whole tumor population or the subpopulation of tumor cells with active GSC promoters CD133 or OCTA4/SOX2. Moreover, administration of GCV would allow the selective killing of replicating cells with active CD133 or OCTA4/SOX2 promoters.

Dually labeled U87 tumor cells were grown either in adherent plates or in non-adherent conditions to form tumorspheres and monitored by BLI. Quantification of images and evaluation of the RLuc/PLuc ratio, a measure of reporter-specific expression relative to cell number, showed significant increases in the activity of CD133 (P = 0.0015) and OCT4/SOX2 (P = 0.0006) promoters when cells were grown as tumorspheres (Fig. 1B), supporting their use as stem cell markers.

To verify the functionality of the tTK gene, U87 cells grown in adherent plates or under tumorsphere forming conditions were treated with 4 μg/ml GCV for a 10-day period, during which PLuc activity was monitored. In both conditions, treatment with GCV resulted in a significant decrease in cell number, as compared with non-treated cells (Fig. 1C−F).

### GCV treatment *in vitro* selects/induces a non-proliferating population of GSCs

While GCV treatment *in vitro* directly targets dividing CD133+ and OCT4/SOX2+ U87 cells, other replicating neighboring tumor cells can also be indirectly killed by a bystander effect. In our *in vitro* tumorsphere experiments, GCV treatment did not eliminate all the cells in culture but left a pool of U87 cells that remain alive after the treatment. Quantification of the fraction of RFP positive cells before and after GCV treatment demonstrated an increase in the proportion of CD133 (P = 0.0003) and OCT4/SOX2 (P = 0.0002) positive cells relative to the total tumor cell population (Fig. 1G,H), a fact that was also accompanied by an increase in the RLuc/PLuc ratio (P = 0.05 and P = 0.0022, respectively) (Fig. S1). Since GCV is toxic for replicating cells, these observations strongly suggest the existence *in vitro* of a CD133+ and OCT4/SOX2+ pool of GCV surviving tumor stem cells.

To further confirm this hypothesis, replication of RFP negative and positive cells in tumorspheres was analyzed using an independent procedure before and after 10 days of GCV treatment (Figs 1I,J and S2). Our results showed that the pool of CD133 and OCT4/SOX2 RFP expressing U87 cells was essentially insensitive to GCV treatment and it comprised a very low proportion compared with that of replicating cells. Conversely, there was a significantly larger proportion of replicating cells within the RFP negative pool and this pool was effectively reduced by GCV treatment.

### Targeting GSCs for GCV mediated cytotoxicity inhibits tumor growth *in-vivo*

A group of 40 SCID mice were stereotactically injected with U87-eGFP-PLuc/CD133-RLuc‐RFP‐tTK (U87-G-P/CD133-R-R-tTK) (n = 20) or U87-eGFP-PLuc/OCT4/SOX2-RLuc‐RFP‐tTK (U87-G-P/OCT4/SOX2-R-R-tTK) cells (n = 20). Starting at day 11 post-implantation, half of the mice in each group (n = 10) were either treated with GCV or not (control) until the end of the experiment. Mice were imaged weekly to monitor tumor growth (PLuc activity) and CD133 or OCT4/SOX2 expression (RLuc activity).

**Figure 2:**
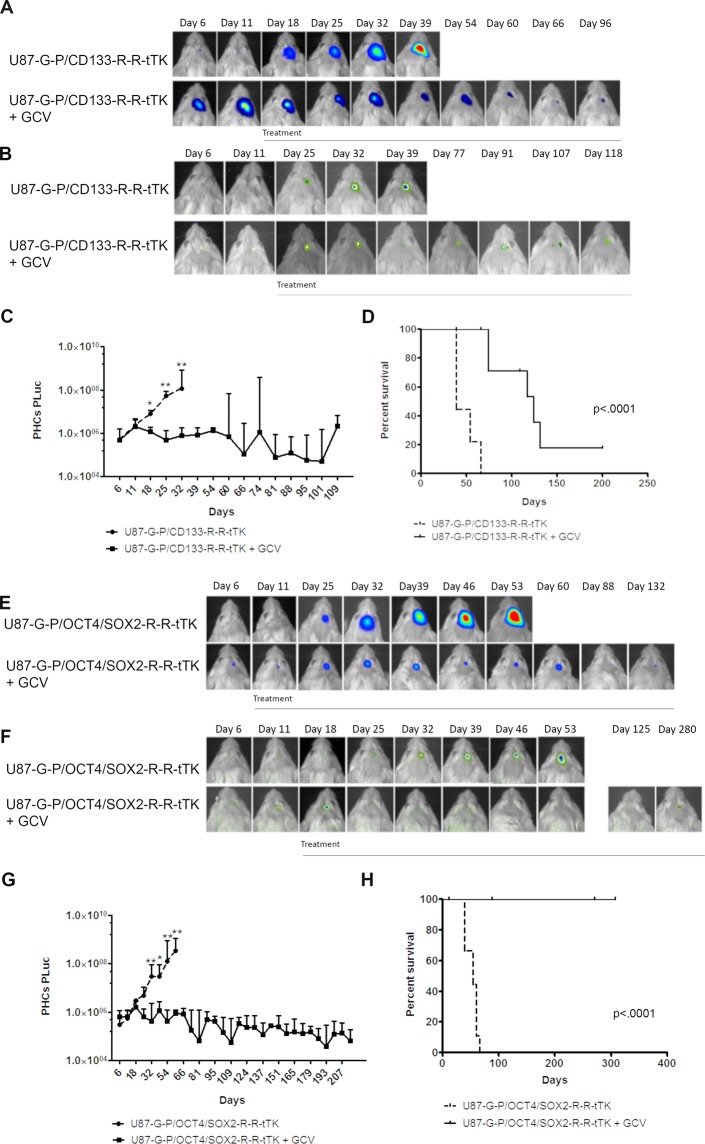
Targeted elimination of CD133+ or OCT4/SOX2+ GSCs abolishes U87 tumor growth *in vivo*. **(A**,**E)** and **(B**,**F)** Pseudo-color BLI images of representative mice showing intensity of PLuc and RLuc activity, respectively, in response to GCV treatment. BLI images are superimposed on black & white images of the corresponding animal; **(C**,**G)** Graphs showing changes in tumor PLuc activity in response to GCV targeting of CD133 (**C**) or OCT4/SOX2 (**G**) positive cells; PHCs, Photon counts; Data points represent interquartile range of the median, *P < 0.5, **P < 0.01; n = 10/group. **(D**,**H)** Kaplan-Meier survival graphs of mice injected with U87-G-P/CD133-R-R-tTK (D) or U87-G-P/OCT4/SOX2-R-R-tTK (H) (P < 0.0001 in both cases).

Images of a representative mouse for each group (Fig. 2A,B,E, F) and Plots of total light events (PHCs) recorded in the acquired images (Fig. 2C,G) showed that GCV targeting of tumor stem cells resulted in a significant inhibition of tumor growth as compared to non-treated controls. In agreement with this, Kaplan Meier graphs (Fig. 2D,H) showed significant increases in animal survival in response to GCV treatment (P < 0.0001 for both experiments). GCV treatment extended median survival of U87-G-P/CD133-R-R-tTK injected animals from 39 days (control group) to 124 days (treated group), while in the case of U87-G-P/OCT4/SOX2-R-R-tTK injected mice, the median survival for the control group was 54 days and treated animals remained alive 1 year after tumor implantation.

In spite of the increase in median survival, neither PLuc nor RLuc activity was completely eliminated by GCV treatment. A stable and measurable non-zero baseline of luciferase activity persisted even after prolonged GCV treatment, emphasizing the existence of a pool of tumor cells that survives the therapy and did not proliferate. The GCV surviving pool comprised cells that expressed RLuc regulated by GSC specific promoters CD133 and OCT4/SOX2 (Fig. 2B,F) respectively.

**Figure 3:**
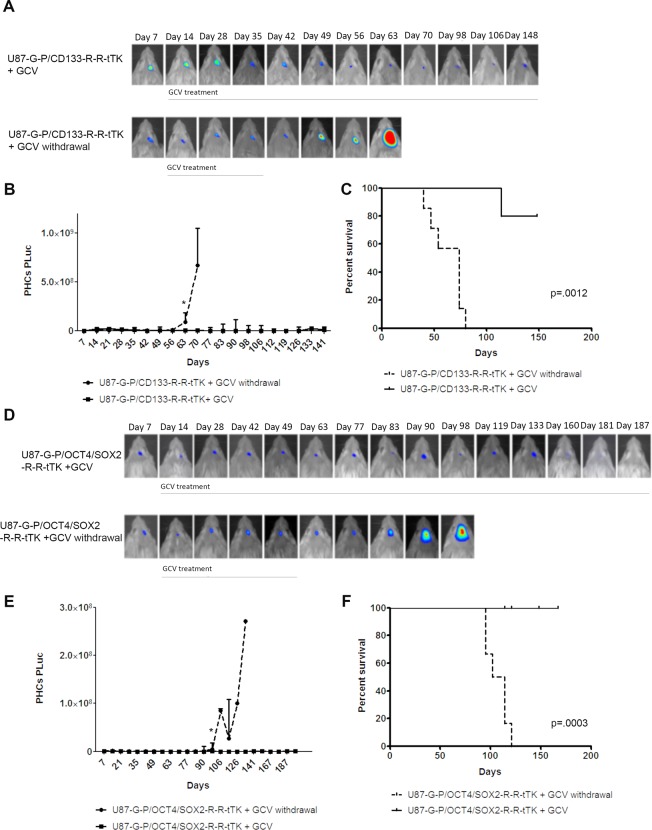
Release of GCV pressure result in tumor relapse *in vivo*. Mice bearing U87-G-P/CD133-R-R-tTK or U87-G-P/OCT4/SOX2-R-R-tTK were treated with GCV during 39 and 54 days, respectively, after which, treatment was withdrawn from half of the animals and maintained in the rest. Tumor growth was monitored by BLI. **(A**,**D)** Pseudo-color BLI images from a representative mouse in each of the experimental groups. BLI images were superimposed on black & white images of the corresponding animal. **(B**,**E)** Graphs show changes in PLuc activity from U87 cells. Data points represent interquartile range of the median; n = 5/group. *P < 0.5. **(C**,**F)** Kaplan-Meyer graphs showing survival of mice, U87-G-P/CD133-R-R-tTK (P = 0.0012) and U87-G-P/OCT4/SOX2-R-R-tTK (P = 0.0003).

In order to determine if GCV treatment had effectively eliminated all tumor-initiating cells or, alternatively if remaining cells could restart tumors, mice were inoculated with U87-G-P/CD133-R-R-tTK or U87-G-P/OCT4/SOX2-R-R-tTK cells and treated or not with GCV (n = 5/group). When mice arrive at the median survival day (determined for non-treated animals at days 39 and 54 for U87-G-P/CD133-R-R-tTK and U87-G-P/OCT4/SOX2-R-R-tTK tumors, respectively), GCV administration was withdrawn in half of the mice of each group. Monitoring of PLuc activity showed an increase in tumor growth by days 63 (CD133) and 77 (OCT4/SOX2), while tumor growth remained inhibited in animals under continued GCV treatment (Fig. 3A,B,D,E). Kaplan Meier survival graphs of U87-G-P/CD133-R-R-tTK and U87-G-P/OCT4/SOX2-R-R-tTK injected mice (Fig. 3C,F) showed that the median survival time was significantly superior in the animals that received GCV daily (P = 0.0012, P = 0.0003, respectively).

Thus, similarly to the *in vitro* model experiments, *in vivo* therapy targeting proliferating GSCs promotes the survival of a quiescent, stem-like luciferase-expressing cell pool capable of reproducing the tumor upon release of the therapeutic pressure.

### Imaging of the GCV surviving GSC niche

To further understand the therapy mechanism, we used the CLARITY procedure to remove light dispersing myelin lipids from fixed brain tissue rendering it transparent to light. This approach allows confocal microscope 3D-imaging of fluorescent tumor cells embedded in the transparent brain tissue.

**Figure 4:**
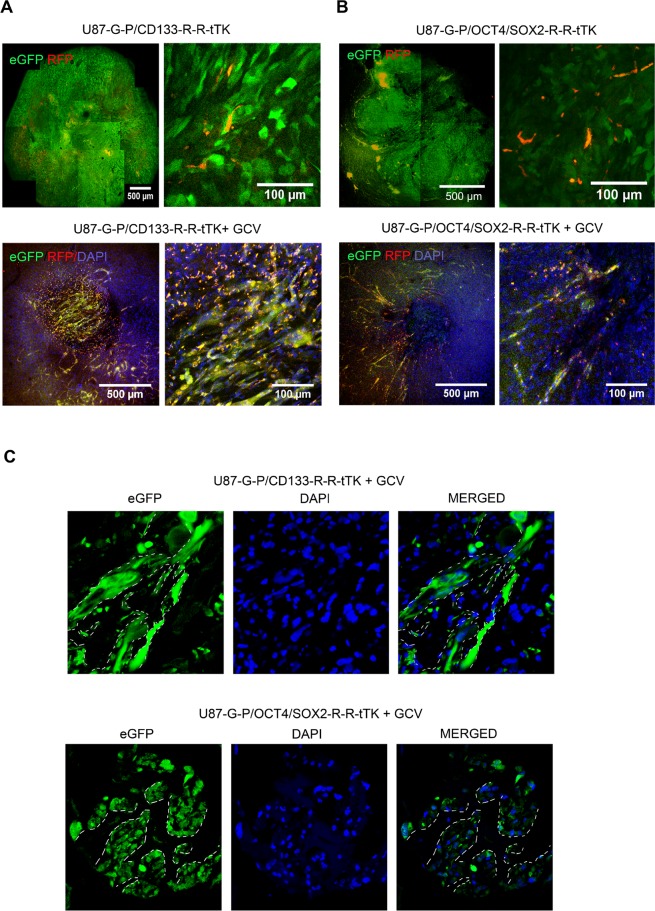
GCV treatment leads to a reservoir of therapy-survivor tumor cells organized in vascular-like structures that express GSC markers. Confocal microscope images of CLARITY treated brain tissue show the U87 tumors cells green fluorescent, and CD133 or SOX2/OCT4 positive cells yellow, resulting from superimposition of red and green fluorescence. **(A**) U87-G-P/CD133-R-R-tTK tumors, untreated (top row) or GCV treated (bottom row); Top and bottom right images are higher magnifications of corresponding left images. **(B**) U87-G-P/OCT4/SOX2-R-R-tTK tumors, untreated (top) or GCV treated (bottom row). Top and bottom right images are higher magnifications of corresponding left images; **(C)** Confocal images of frozen tumor sections from U87-G-P/CD133-R-R-tTK and U87-G-P/OCT4/SOX2-R-R-tTK treated with GCV. White lines highlight vascular-like structures that remain after therapy. Blue stain shows cell nuclei.

Confocal images of U87-G-P/CD133-R-R-tTK or U87-G-P/OCT4/SOX2-R-R-tTK tumors following the CLARITY procedure showed red fluorescent tumor stem cells forming dispersed aggregates within a complex structure comprising green fluorescent tumor cells (Fig. 4A,B, top panels).

In the case of GCV-treated U87-G-P/CD133-R-R-tTK or U87-G-P/OCT4/SOX2-R-R-tTK tumors, the CLARITY procedure allowed us to visualize small, approximately 500 µm in diameter, niches of therapy-surviving tumor cells. Magnification of images from these cell reservoirs revealed their organization into a network of vascular-like structures formed by eGFP tumor cells as well as cells expressing both RFP and eGFP reporters (resulting in yellow fluorescence); thus, putative tumor stem cells (Fig. 4A,B, bottom panels).

Conventional immunohistochemical analysis of the treated tumors confirmed that after GCV treatment, the remaining small tumor area consisted of cellular structures and voids reminiscent of capillary networks (Fig. 4C). We thus propose that in response to anti-proliferative treatment, the regeneration capacity of a tumor resides in a surviving niche of specialized, non-replicating tumor cells that express the CD133 and OCT4/SOX2 stem cell markers and form tubular-like structures.

**Figure 5:**
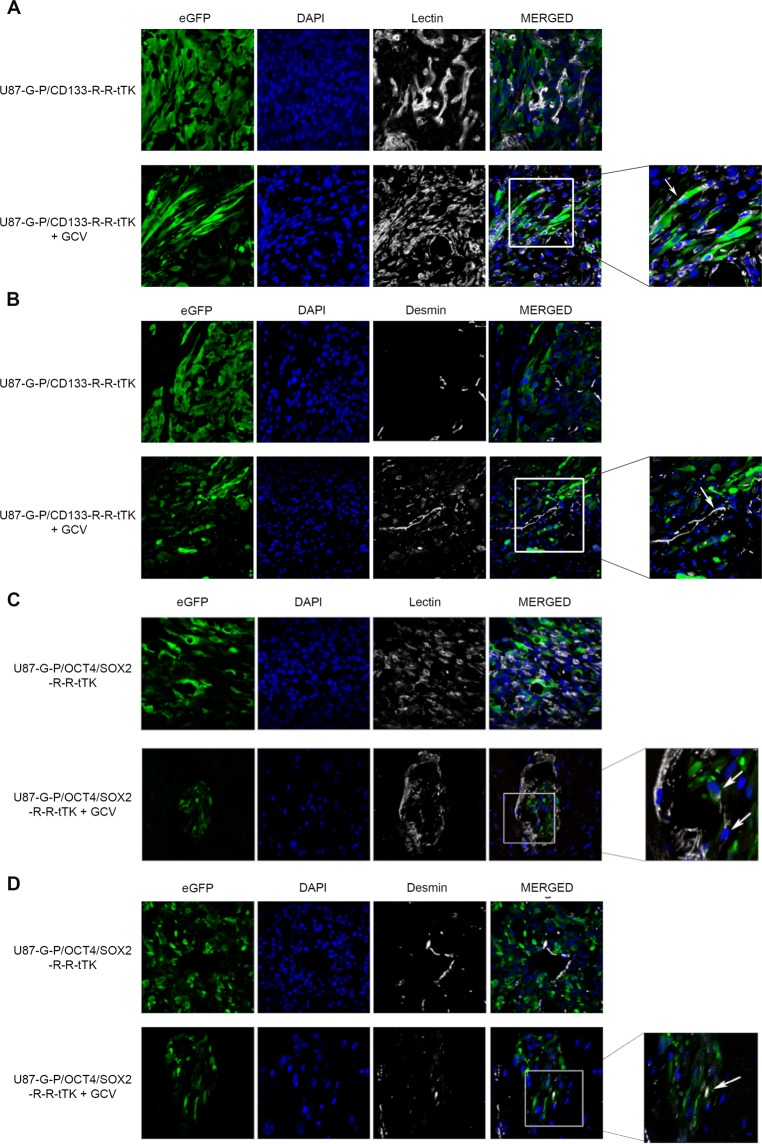
Tumor cells surviving GCV treatment rarely show endothelial or pericyte markers. Brains of mice bearing tumors that had been treated or not with GCV were harvested, fixed and analyze by confocal microscopy. **(A**,**B)** U87-G-P/CD133-R-R-tTK tumor counterstained with DAPI (nuclei) and with either GSLI B4 isolectin (A) (endothelial cell marker) or desmine (**B**) (pericytic cell marker). **(C**,**D)** U87-G-P/OCT4/SOX2-R-R-tTK tumor analyzed as in (**A**,**B**). Rare instances of colocalization of green fluorescent tumor cells and lectin (**C**) or desmine (**D**) positive cells are indicated by white arrows.

To determine whether the tubular structures found after GCV treatment were tumor cells differentiated into endothelial cells or pericytes, we analyzed them using immunohistochemistry procedures for the presence of specific lineage markers. However, we rarely observed colocalization of eGFP-expression and the specific endothelial or pericytic cell markers lectin and desmin, respectively (Fig. 5A,C and Fig. 5B,D); instances of isolated eGFP positive tumor cells that also expressed lectin or desmin are shown in Fig. 5A–D (white arrows).

### The capacity of differentiated tumor endothelial cells and pericytes to recapitulate tumor growth

The remarkable therapy resistant tubular-like structures suggest the possibility that cell types with GSCs properties may be preexistent within the endothelial and/or pericytic compartments of the tumor, and are selected or enriched for by the therapeutic pressure. Alternatively, the tubular structures may be remnants of vasculogenic mimicry, a process whereby tumor cells can form fluid-conducting channels mimicking a vascular function, being in this case enriched in GSCs as a result of therapy.

In order to verify these hypotheses, and its generality, SCID mice, 20 per group, were inoculated with either U87-eGFP-PLuc, NCH644-eGFP-PLuc or NCH421k-eGFP-PLuc cells (the latter two are aggressive diffusely growing tumors, see Fig. S3) and allowed to grow. When the tumor reached the appropriate size; tumors were harvested and disaggregated into individual cells. Cells were sorted for simultaneous expression of eGFP (tumor cells) and two different endothelial cell markers (CD31 and CD105) and collected as double positive or double negative cell pools. Alternatively, cells were sorted for the expression of eGFP and two pericyte markers (CD146 and CD248) and also collected into double positive and double negative cell pools. The four types of cell pools were grown under tumorsphere forming conditions to evaluate their tumorsphere generating capacity.

The first relevant observation was that double negative and double positive cell pools were capable of producing tumorspheres.

To evaluate tumor-forming capacity, tumorsphere cells (1 × 10^5^ cells/mouse) were injected in SCID mice (n = 6) and tumor growth was monitored weekly by BLI.

*In vivo* results showed that, regardless of the tumor type of origin, tumorspheres from both CD31+ CD105+ cells and CD146+ CD248+ cells were able to generate tumors that killed the host.

**Figure 6:**
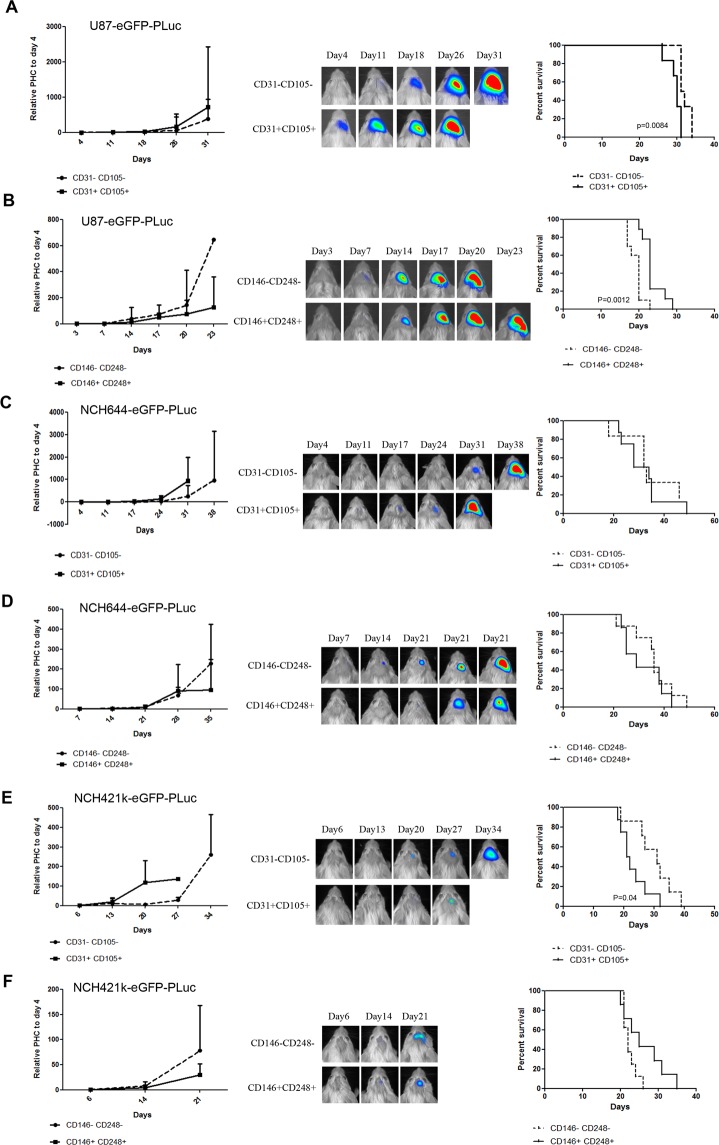
Tumor cells positive for endothelial and pericyte markers were selected by FACS, grown under tumorsphere conditions and implanted in the brain of SCID mice to monitor tumor development. **(A**–**F**, right panel) Graphs displaying differences in growth of tumors derived from U87 (**A**,**B**), NCH644 (**C**,**D**) or NCH421k (**E**,**F**) tumorsphere cells. (**A**,**C** and **E**) show differences between double positive endothelial markers and the depleted control. (**B**,**D** and **F**) show differences between double positive pericyte markers and the depleted control; data points represent standard deviation of the mean related to the firs day; n = 6/group. **(A**–**F** middle panel**)** Pseudo-color BLI images from a representative mice in each of the experimental groups implanted with tumorsphere cells from positive endothelial (**A**,**C** and **E**) or positive pericyte (**B**,**D** and **F**) selected cells, respectively; BLI images were superimposed on black and white images of the corresponding mice. **(A**–**F** left panel) Kaplan-Meier graphs showing survival differences between mice implanted with depleted controls and corresponding double positive endothelial or pericyte lineage.

As expected, tumorspheres from cell pools negative for either endothelial or pericytic markers that also comprise “non-vascular” GSCs, also recapitulated tumors and killed their hosts (Fig. 6A–F, left and middle panels).

Kaplan Meier plots of animal survival show no statistical difference in the capacity of tumors from the different tumorsphere types to kill mice.

Thus, these data indicate that both, double positive cells of endothelial and pericyte lineages derived from tumors are able to dedifferentiate and recapitulate tumors, pointing at the tumor vascular system as a reservoir of tumor-initiating cells.

Moreover, it appears that the abundance of tumor cells with the capacity to recapitulate tumors is similar in the tumor vascular compartment (endothelial and pericytic lineages) and the rest of the tumor.

## Discussion

In previous work we observed that cytotoxic tTK expressing hAMSCs that targeted the GSC niche differentiated to vascular components in close proximity to CD133 expressing GBM cells and were capable of reducing tumor burden by a factor or 10^4^ in the presence of GCV. These results led us to speculate that cell therapy was targeting the GSC niche. However, even after prolonged treatment, release from the therapeutic pressure would result in tumor recurrence^31^.

In the current work, we test if a therapeutic strategy directly targeting GSCs could eliminate a GBM U87 tumor completely. We designed a strategy to express in all tumor cells PLuc and eGFP reporters and, RLuc, RFP and tTK genes in the subpopulation of GSCs with active CD133 or OCTA4/SOX2 promoters. In this manner, the tTK gene would allow us to selectively “kill at will” replicating tumor cells that activated the GSC specific promoters. CD133 and OCT4/SOX2 promoter functionality and responsiveness were validated by demonstrating *in vitro* cell killing capacity in the presence of GCV and production of renilla luciferase activity in response to induction of stemness in tumorspheres.

Following we implanted the targeted cells in live animal brains and the resulting tumors were subject to GCV therapy. In the *in vivo* experiments, this strategy was very effective at reducing tumor growth. However, it should be noted that targeting tumor cells with an active OCT4/SOX2 promoter had a more potent effect than targeting those with an active CD133 promoter; probably due to the higher frequency of OCT4/SOX2 active cells in our GBM model. Interestingly, the therapeutic effectiveness of targeting GSCs was comparable to that of using tTK expressing hAMSCs in previous work^31^.

Also, as in the case of hAMSC therapy, neither one of the GSC targeting strategies was capable of completely eliminating tumors, which in the current case survived during long treatment periods at nearly undetectable levels, as would be indicated by background levels of PLuc and CD133 and SOX2/OCTA4 regulated RLuc activities. Release of the therapeutic pressure resulted invariably in the increase of luciferase activity, growth of the tumors and death of the animals.

Since the TK/GCV strategy is toxic for replicating cells, we concluded that our therapy might be selecting or inducing a stable, possibly non-replicating pool of GSCs expressing CD133 and OCT4/SOX2 regulated reporters that survived to the cytotoxic agent; a conclusion borne out by our *in vitro* experiments.

Using the CLARITY procedure in combination with confocal microscopy we were able to observe a scattered distribution of red-fluorescent GSCs within the 3D organization of green-fluorescent tumor cells in undisturbed tumors. Surprisingly, in GCV treated animals, we found very small, therapy-resistant structures made of networks of tubular-like green fluorescent cells also expressing the red fluorescent GSC tracer.

While it is widely accepted that GBM cells can transdifferentiate into endothelial cells^26,27,32^ or pericytes^28^, except for very infrequent instances, we failed to immunohistochemically detect endothelial and pericyte markers in the therapy-resistant structures. Thus, suggesting that expression of GSC characteristics could have arisen from “dedifferentiation” of tumor endothelial or pericytic cells.

An alternative explanation could be related to vasculogenic mimicry, a mechanism by which tumor cells form functional vascular-like structures without vascular differentiation^33,34^. In such context, vascular mimicking structures could be a reservoir in which special non-dividing GSCs would become enriched as dividing tumor cells are killed by therapy.

The therapy resistant tubular structures suggested that the tumor vascular compartment could be a, so far undetected, reservoir of GSCs. To explore such hypothesis, we use U87 and 2 other glioblastoma cell lines NCH644 and NCH421k that were isolated from brain tissue and were cultured in serum-free spheroids, making them a good model of glioblastoma explants. We isolated from growing tumors of the 3 different cell lines, green fluorescent tumor cells that simultaneously expressed two endothelial cell markers (CD31 and CD105) or two pericyte markers (CD146 and CD248) and used them, as well as the corresponding depleted cell pools, to produce tumorspheres. Our *in vitro* tests indicated that, as expected, the tumor cell pools depleted of endothelial or pericytic markers retain the capacity to produce tumorspheres. Surprisingly, cells double positive for two endothelial or two pericyte markers were also capable of producing tumorspheres. Moreover, tumorspheres from the endothelial and pericytic compartments of the three tumor types tested were as capable to produce mice killing tumors as those derived from the depleted controls representing the rest of the tumor.

Our findings suggest reasons for the failure of some therapeutic strategies. We provide first evidence showing that some cytotoxic therapies targeting replicating tumor-initiating cells can dramatically reduce tumor burden but fail to eradicate tumors, inducing instead the formation of vascular-like structures comprising therapy-surviving tumor-initiating cells that will recapitulate the tumor. Moreover, tumor cells positive for endothelial and pericytic markers form a compartment that comprises tumor cell types that are or can be induced to form tumor-initiating cells with the capacity to recapitulate tumors.

## Experimental Procedures

### Lentiviral vector constructs

*CMV-PLuc-IRES-eGFP vector:* Lentiviral construction CMV‐PLuc‐IRES‐eGFP was kindly provided by Dr. Trono^35^. The vector comprised PLuc under control of the CMV promoter upstream from the IRES for expression of the eGFP. *CMV-RLuc-RFP-tTK lentiviral vector:* Lentiviral construct CMV-RLuc-RFP-tTK comprises the coding sequences for the trifunctional chimeric multiprotein RLuc reporter, RFP, and tTK under transcriptional control of the CMV promoter, a kind donation from Dr. S.S Gambhir (Stanford, CA)^36^. *CD133-RLuc‐RFP‐tTK:* The pGL3enh-P1 CD133 expression plasmid was kindly provided by Dr. Shinya Tanaka (Hokkaido University Graduate School of Medicine, Sapporo, Japan)^37^. We have amplified the promoter region and cloned into CMV-RLuc-RFP-tTK vector. See details in Supplementary Material. *OCT4/SOX2-RLuc‐RFP‐tTK:* We used the plasmid PL-EOS-C(3+)-eGFP-IRES-PuroR (from James Ellis, Addgene plasmid # 21313) containing 3 consecutive repeats of the SOX2/OCT4 binding sites to generate the OCT4/SOX2-RLuc‐RFP‐tTK reporter gene construct. See details in the Supplementary Material.

### Cell culture

Human GBM cells U87 (ATCC HTB-14) and human embryonic kidney cells 293T (ATCC, CRL-11268™) were grown in nutrient mixture DMEM/Hams F12 or DMEM-hg (Sigma) respectively, containing 10% heat-inactivated FBS (Sigma), 2 mM L-glutamine (Sigma) and 50 units/ml penicillin/streptomycin (Sigma). NCH644 and NCH421k (CLS, cell line services) were grown in GBM-MG, ready to use (CLS, cell line services) in T75 non-adherent plastic flasks.

### Lentiviral particle production and cell infection

Lentiviral particles were produced by transfecting HEK293T cells with pMD-G-VSV-G, pCMV DR8.2 and either CMV-PLuc-IRES-eGFP, CD133-RLuc-RFP-tTK or OCT4/SOX2-RLuc-RFP-tTK respectively to HEK293T cells. U87, NCH644, and NCH421K cells were infected with CMV-PLuc-IRES-eGFP viral stock and expression of the green fluorescent protein were used to select positively transduced cells by flow cytometry (Area Fusion Cell Sorter) obtaining U87-eGFP-PLuc, NCH644-eGFP-PLuc or NCH421k-eGFP-PLuc. Following, U87 eGFP positive cells were infected with either CD133-RLuc-RFP-tTK or OCT4/SOX2-RLuc-RFP-tTK viral stocks to obtain U87-G-P/CD133-R-R-tTK or U87-G-P/OCT4/SOX2-R-R-tTK respectively. See details in the Supplementary Material.

### Tumorsphere culture

U87-G-P/CD133-R-R-tTK and U87-G-P/OCT4/SOX2-R-R-tTK cells were cultured in T75 non-adherent plastic flasks with serum-free DMEM-hg supplemented with 20 ng/μl of Epidermal growth factor (EGF, PeproTech); 10 ng/μl of Fibroblast growth factor-2 (FGF-2, PeproTech); B27 supplement (Invitrogen) and N2 supplement (Invitrogen).

### Bioluminescence Imaging and determination of luciferase activity

*In vitro cultures:* Tissue culture plates were imaged using an ImagEM X2 C9100-23BEM-CCD (Hamamtsu Photonics) cooled at −80 °C. See details in the Supplementary Material. *In vivo* BLI of SCID mice was performed as described previously^38^. See details in the Supplementary Material.

### *In Vitro* promoter activation assay

5 × 10^4^ U87-G-P/CD133-R-R-tTK or U87-G-P/OCT4/SOX2-R-R-tTK cells were seeded in 24-well plates and grown as adherent cells or as tumorspheres for 3 weeks. Cells were imaged immediately following addition of PLuc or RLuc substrate reagent. RLuc/PLuc ratio was used to measure changes in gene-specific expression relative to cell number.

### *In Vitro* GCV treatment

U87-G-P/CD133-R-R-tTK or U87-G-P/OCT4/SOX2-R-R-tTK cells were grown in plates as adherent cells or as tumorspheres for 3 weeks. Then, 5 × 10^2^ cells were seeded in a 96-well plate (sextuplicates for each condition) and treated or not with GCV (4 µg/ml; Cymevene, Roche) for 10 days. Medium was replaced every 3 days. BLI images were acquired at the indicated times.

### Flow cytometry analysis

U87-G-P/CD133-R-R-tTK or U87-G-P/OCT4/SOX2-R-R-tTK cells were grown in tumorsphere forming conditions for 3 weeks. Then, half of the cells were cultivated with GCV (4 µg/ml). After 10 days of treatment, cells were analyzed by flow cytometry to determine the fraction of RFP positive cells (Area Fusion). Histograms were analyzed using the Flowing Software-2. The proliferation of the RFP negative and positive cells, before and after the GCV treatment, was quantified using the Click-iT® Plus EdU Pacific Blue™ Flow Cytometry Assay Kit (Molecular Probes) according to the manufacturer’s instructions. The percentage of cells in replication was analyzed by flow cytometry.

### Animal experiments

Adult 6–8 weeks old SCID mice (Harlan Laboratories) were kept under pathogen-free conditions in laminar flow boxes. Animal maintenance and experiments were performed in accordance with established guidelines of the Catalan *Direcció General del Medi Natural*. The experimental protocol was approved by the Government of Catalonia, protocol num. 4565. See details in Supplementary Material.

### Clarity

Brain samples were cleared for 3D imaging following the CLARITY procedure^39^. Briefly, mice were sacrificed by perfusion with acrylamide/paraformaldehyde. Fixed brains were harvested and thick-sectioned in 500 µm thick slices. Hydrogel-brain slices were cleared by passive lipid diffusion and mounted for tri-dimensional microscopy analysis of intact-cleared tumor tissues using a Leica TCS-SPE confocal fluorescence microscope. Microscope images were reconstructed and analyzed using ImageJ/Fiji and Imaris software. See details in the Supplementary Material.

### Inmunohistochemistry

Brains were harvested, fixed, embedded in OCT (Sakura) and snap-frozen in liquid nitrogen-cooled isopentane. Serial 10 μm thick cryosections of brain tissue were prepared for immune detection as described in the Supplementary Materials.

### Isolation of pericytes and endothelial cells from GBM tumors

Experimental animals, 20/group, were each implanted in the brain with 1 × 10^5^ U87-eGFP-PLuc, NCH644-eGFP-PLuc or NCH421k-eGFP-PLuc cells. Tumors were extracted and mechanically disaggregated using a scalpel. Single cells were separated from tissue clumps using a cell strainer and incubated with red blood cells lysis buffer during 5 minutes. Individual eGFP expressing tumor cells positive for pericytic and endothelial cell markers were isolated using a fluorescence-activated cell sorter (FACS). Pericyte-like cells were sorted from total cells by selecting eGFP+/CD146+/CD248+ cells with anti CD146-VioBlue (Miltenyi Biotec) and anti CD248-Alexa Fluor 647 (clone B1/35 Beckton Dickinson). Endothelial-like cells were sorted by selecting eGFP+/CD31+/CD105+ cells with anti CD31-APC (Miltenyi Biotec) and CD105-VioBlue (Miltenyi Biotec). Selected positive cells of either type were grown in non-adherent plates with tumorsphere culture media.

### Statistical analysis

StataSE12 software was used for the statistical analysis of BLI data. T-test was applied to compare 2 groups that adjust to a normal curve. When data did not adjust to a normal curve or was not possible to normalize Two-sample Wilcoxon rank-sum (U-Mann-Whitney) test was used. Kaplan-Meier survival analyses were performed using GraphPad Prism 5 Software. Resulting plots were compared by the Log-rank (Mantel-Cox) test. Statistically significant differences were considered when P < 0.05.

## Supplementary information

Supplementary information

## Data Availability

No datasets were generated or analyzed during the current study.
