# Supplementary material for: Targeting of replicating CD133 and OCT4/SOX2 expressing glioma stem cells selects a cell population that reinitiates tumors upon release of therapeutic pressure
Source: Sci Rep. 2019 Jul 2;9:9549. doi: 10.1038/s41598-019-46014-0 (PMC6606606; doi:10.1038/s41598-019-46014-0)
Supplement: Supplementary file 1 — Supplementary information [file 41598_2019_46014_MOESM1_ESM.pdf]

Targeting of replicating CD133 and OCT4/SOX2 expressing glioma stem cells selects a cell population that reinitiates tumors upon release of therapeutic pressure

Marta Guerra-Rebollo, Cristina Garrido, Lourdes Sánchez-Cid, Carolina Soler-Botija, Oscar Meca-Cortés, Nuria Rubio, Jerónimo Blanco.

Supplementary Figures:

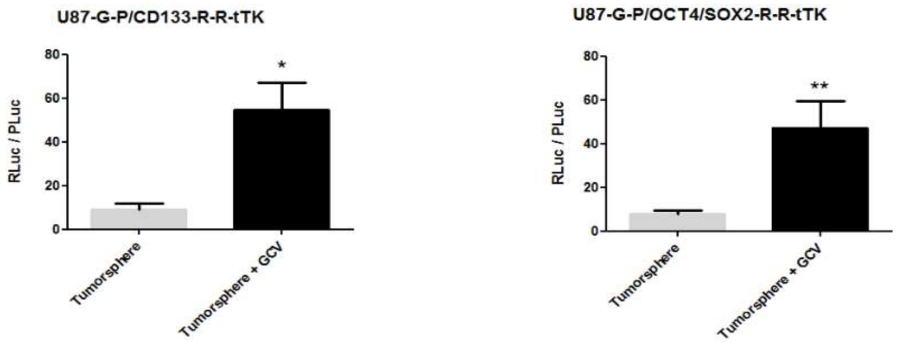

Supplementary Figure S1. GCV treatment produces an increase in RLuc/PLuc activity ratio. U87-G-P/CD133-R-R-tTK or U87-G-P/OCT4/SOX2-R-R-tTK cells were grown under tumorsphere forming conditions in presence or absence of GCV during 10 days and analyzed by BLI on the 10th day to determine activity from both luciferases. Histograms show the ratio of RLuc/PLuc activity calculated from BLI images. Histograms represent the average of six wells; bars represent the standard deviation of the mean; * P<.5, ** *P*<.01.

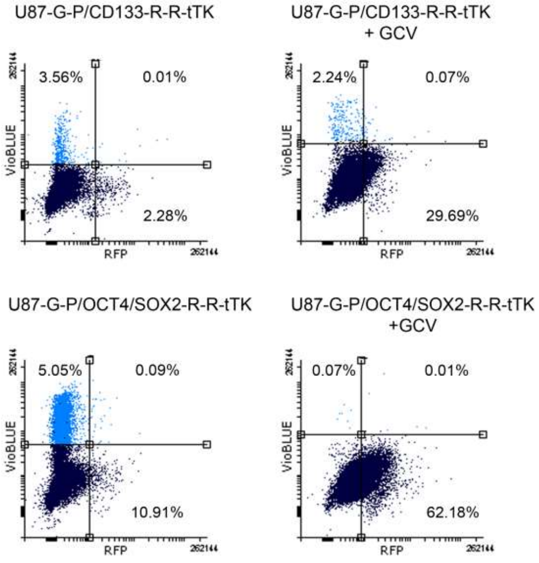

Supplementary Figure S2. The reduction in replicative cell number after GCV treatment affects predominantly the RFP negative cell population. Histograms showing the reduction in the number of replicative cells (VioBlue positive) in the RFP negative and positive populations. Replicating cells are highlighted in light blue.

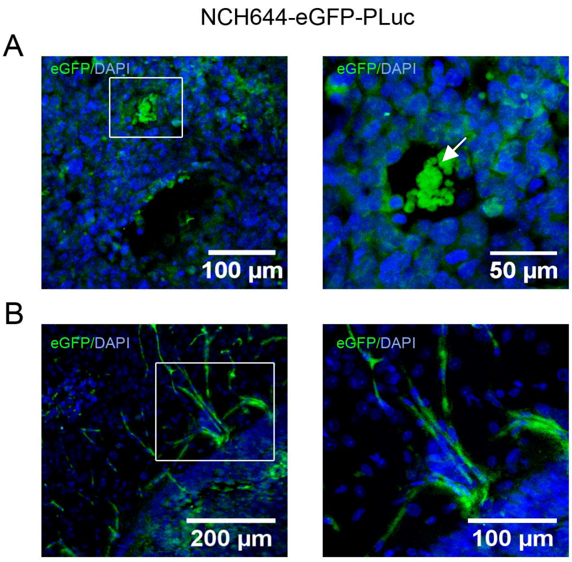

Supplementary Figure S3. Confocal microscope images of CLARITY brain tissues show the NCH644 tumors cells green fluorescent. **(A)** NCH644 tumors form functional vascular-like structures, the amplified box (right panel) show a tumoral vascular structure with erythrocytes inside (white arrow). **(B)** NCH644 tumoral cells can spread from the primary tumor, the amplified box in the right panel exhibit some tumoral cells advancing into the brain tissue.

SUPPLEMANTARY METHODS

Lentiviral vector constructs

CD133-RLuc-RFP-tTK: To generate the CD133-RLuc-RFP-tTK lentiviral construct, the promoter region of the CD133 gene was amplified by PCR using forward primer: 5′-GGACTAGTACAATTATGGTGGAAGAAGG-3' and reverse primer: 5′-GGACTAGTGTGGGGATCTGCCTCAGTC-3' (*Spe*I sites are underlined). The PCR products were gel-purified, digested with SpeI and cloned into hrl-RFP-tTK with *Spe*I sites. Insert orientation was verified by digestion with BamHI. *OCT4/SOX2-RLuc-RFP-tTK:* We used the plasmid PL-EOS-C(3+)-EGFP-IRES-PuroR containing 3 consecutive repeats of the SOX2/OCT4 binding sites to generate the OCT4/SOX2-RLuc-RFP-tTK reporter gene construct. The region with the three (3+) SOX2/OCT4 repetitions was amplified by PCR using the forward primer: 5'-GCTCTAGAAGTGAACGGATCCTCCCTTT-3' and the reverse primer: 5'-AACAGTACCGGAATGCCAAG-3' (a XbaI site is underlined). The PCR products were gel-purified, digested with XbaI and cloned into the *Spe*I sites of lentiviral construct hrl-RFP-tTK

Lentiviral particle production and cell infection

The day previous to transfection, 4×10^6 HEK 293T cells were seeded on 10 cm^2 poly-D-lysine (Sigma) treated plates. 6 µg of each lentiviral transfer vector, were mixed with 2 µg of viral envelope plasmid (pMD-G-VSV-G) and 4 µg of packaging construct (pCMV DR8.2) in 250 µl of 150 mM NaCl. This DNA-NaCl solution were then mixed with 48 µl of 1 mg/ml polyethylene amine (Polyscience) in 250 µl of 150 mM NaCl, and incubated at room temperature (RT) for 30 min. This DNA solution was then added to the plate containing the HEK293T cells plus medium and incubated for 16 h at 37°C with 5% CO_2. The following day, the transfection solution was removed and medium without FBS was added to the cells. Following 48 h incubation, the supernatant was collected, centrifuged at 2000 rpm to remove cell debris, and filtered through a 0,45-µm low-protein binding filter (Corning).

Bioluminiscence Imaging and determination of luciferase activity

In vitro cultures: For BLI of culture plates: medium was removed, the wells were rinsed twice with PBS 1x and imaged immediately following addition of Pluc or Rluc substrate reagent (Luciferin from Regis Technologies or coelenterazine from p.j.k). In order to register the position of the light signal, an additional image was obtained using a white light from a lamp in the detection chamber. Light events recorded in images were calculated using the HokawoTM Imaging Software (Hamamatsu Photonics) and expressed as photon counts (PHC) after subtracting the background. The net number of PHCs in the area of interest was calculated using the formula: PHCs=(total number of PHCs in the area of interest)−[(number of pixels in the area of interest)×(average background PHCs per pixel)]. *In vivo bioluminescent imaging:* Mice were anesthetized i.p. and then injected i.p. with 150 µl of luciferin (Regis Technologies) (16.7 mg/ml in saline) were injected i.p. or through tail vein with 5 µl of coelenterazine (p.j.k.) (3.33 mg/ml) diluted in 165 µl of PBS (final concentration 0.1 mg/ml) were administrated through tail vein. Animals were placed in the detection chamber of a high efficiency ImagEM X2 C9100-23BEM-CCD Imaging System (Hamamatsu Photonics) and images were acquired from the dorsal direction. A second image of the animal was obtained using a white-light source inside the detection chamber, to register the position of the luminescence signal. Quantification and analysis of photons recorded in images was done using the Wasabi image analysis software as described above. Light measurements were expressed as PHCs following subtraction of background as previously described.

Animal experiments

Animals were anesthetized by i.p. injections of 3.3 mg/kg xylazine (Rompun, Bayer DVM) and 1.39 mg/kg ketamine (Imalgene 100, Merial Laboratorios). Subsequently mice were mounted in a stereotactic frame (Stoelting) and their heads secured using a nose clamp and two ear bars. For stereotactic cell implantation, cells were injected at a 0.6 µl/min rate using a Hamilton syringe series 700 at 0.6 mm posterior, 2 mm lateral and 2.75 mm depth in relation to the Bregma anatomical point. The scalp was closed by suture and buprenorphine (0.05 mg/kg)

(Buprecare, Divasa-Farmavic SA) was administrated as analgesic. The treated group received a daily ip dose of 50 mg/kg of GCV (Cymevene, Roche) while the control group received the same volume of PBS daily.

CLARITY

For 3D anatomical mapping of the whole tumour structure, tumour samples were passively cleared using the CLARITY procedure and analyzed by tomographyc fluorescence microscopy. Animals were transcardially perfused with a hydrogel solution comprising: paraformaldehyde (16%), acrylamide (40%)-bisacrylamide (2%) and VA-004 Initiator (0.05%), and the brain was immediately isolated and kept in hydrogel solution at 4°C for 3 days protected from light. The tissue-containing solution was then polymerized at 37°C and the sample was thick-sectioned in 500µm slices using a rodent brain razor blade slicer matrix (Zivic Instruments). Cleared tissue was obtained by passive lipid diffusion in Sodium Dodecyl Sulfate (4%), 200mM Boric Acid (pH 8.5) solution at 45°C, protected from light. Transparent tissue-hydrogels were obtained after four days of incubation. Samples were mounted in 80% glycerol between glass slides and imaged using a Leica TCS-SPE confocal fluorescence microscope. Optically transparent tissue-hydrogel slices were scanned, Z-axis, in 1-5 µm steps through a maximum depth of 200 µm allowing single-cell resolution. Microscopy images were reconstructed and analyzed with the ImageJ/Fiji software.

Inmunohistochemistry

Brains were harvested, fixed with 4% paraformaldehyde (Sigma) during 24 hours, immersed in 30% sucrose in PBS, embedded in OCT (Sakura) and snap-frozen in liquid nitrogen-cooled isopentane. Brain serial cryosections of 10 µm thick were incubated with primary antibodies against Desmin (2 µg/mL) (Abcam) and with biotinylated GSLI B4 isolectin (1:100 dilution) (Vectors Labs). Sections were also incubated with antibodies against RFP (1:50 dilution) (Chromotek) and GFP (5 µg/mL) (Abcam) to enhance the detection of reporter-transduced cells. Secondary antibodies conjugated with Alexa488, Cy3, and Cy5 (1 µg/mL) (Jackson

ImmunoResearch) were used for detection. Nuclei were counterstained with 4,6-diamidino-2-phenylindole (DAPI, Sigma) and the results were analyzed using a laser confocal microscopy (Axio-Observer Z1, Zeiss).
